# Usability and feasibility of a wearable sensor-based monitoring system providing feedback on sedentary behaviour during inpatient and home-based geriatric rehabilitation

**DOI:** 10.1007/s41999-026-01616-6

**Published:** 2026-10-06

**Authors:** Suzanne M. Debeij, Miriam L. Haaksma, Margot W. M. de Waal, Jolanda C. M. van Haastregt, Margriet C. Pol, Marise J. Kasteleyn, Eléonore F. van Dam van Isselt

**Affiliations:** 1 Department of Public Health and Primary Care, Leiden University Medical Center, Leiden, The Netherlands; 2 University Network for the Care Sector South-Holland, Leiden University Medical Center, Leiden, The Netherlands; 3 Department of Health Services Research, Faculty of Health Medicine and Life Sciences, CAPHRI Care and Public Health Research Institute, Maastricht University, Maastricht, The Netherlands; 4 Limburg Living Lab in Ageing and Long-Term Care, Maastricht University, Maastricht, The Netherlands; 5 Department of Medicine for Older People, Amsterdam University Medical Center, Location Vrije Universiteit Amsterdam, Amsterdam, The Netherlands; 6 Amsterdam Public Health, Ageing & Later Life, Amsterdam, The Netherlands; 7 Amsterdam University of Applied Sciences, Research Group Occupational Therapy: Technology and Participation, Faculty of Health, Centre of Expertise Urban Vitality, Amsterdam, The Netherlands; 8 University Network of Organizations for Care for Older Adults, Amsterdam University Medical Center, Amsterdam, The Netherlands; 9 National eHealth Living Lab, Leiden, The Netherlands

**Keywords:** eHealth, Digital health technologies, Post-acute care, Elderly, Wearable, Feedback

## Abstract

**Objective:**

This study explored the usability and feasibility of a sensor-based monitoring system that provides feedback on sedentary behaviour during inpatient and home-based geriatric rehabilitation from the perspectives of patients and healthcare professionals.

**Methods:**

This mixed-methods study, which is part of the Better@Home study, included a focus group with care professionals, patient surveys, and collection of activity data (e.g. sedentary time and bout duration) via a wearable sensor monitoring system. Usability was assessed qualitatively, while feasibility was assessed using both qualitative data and quantitative adherence to the monitoring system. Focus group data were analysed using the framework method; other data were analysed descriptively.

**Results:**

Twenty-six patients and nine care professionals participated. Usability was perceived as limited due to challenges on both the technology (e.g. measurement accuracy) and implementation level (e.g. tailored use and added value), which impeded integration into existing workflows. Feasibility was deemed sufficient since adherence levels were high.

**Conclusions:**

From the perspectives of patients and healthcare professionals, a sensor-based monitoring system that provides feedback on sedentary behaviour showed sufficient feasibility and limited usability. To facilitate the integration into existing workflows, measurement accuracy should be improved, and implementation should focus on tailored use that offers a clear added value, in line with patient goals.

**Supplementary Information:**

The online version contains supplementary material available at https://doi.org/10.1007/s41999-026-01616-6.

## Key summary points

**Aim:**

To explore the usability and feasibility of a sensor-based monitoring system that provides feedback on sedentary behaviour during inpatient and home-based geriatric rehabilitation from the perspectives of patients and healthcare professionals.

**Findings:**

Usability was perceived as limited due to various concerns, e.g. measurement accuracy. Feasibility was deemed sufficient, but requires practical improvements.

**Message:**

Sensor-based feedback on sedentary behaviour in geriatric rehabilitation should be easy to use, accurate, aligned with patient goals, and supported by the entire multidisciplinary team to ensure usability and feasibility.

**Supplementary Information:**

The online version contains supplementary material available at https://doi.org/10.1007/s41999-026-01616-6.

## Introduction

Geriatric rehabilitation plays a critical role in helping older adults with disabling impairments to regain independence [1]. These older adults often present with conditions such as frailty, multimorbidity, deconditioning, and varying degrees of cognitive impairment. Geriatric rehabilitation improves various outcomes, including functional parameters, and reduces nursing home admissions and mortality [1]. It can be delivered in an inpatient setting, home-based, or through a combination of both, with home-based rehabilitation often serving as a transitional phase [1, 2]. Treatment programmes are personalized and consist of several modules of geriatric rehabilitation.

One of the core elements in rehabilitation is engaging in physical activity to regain functional performance. However, recent studies indicate that patients in geriatric rehabilitation are highly sedentary [3, 4]. Sedentary behaviour is defined as “any waking behaviour resulting in an energy expenditure of ≤ 1.5 Metabolic Equivalent of Task while in a sitting, reclining or lying posture” [5]. The high level of sedentary time persists throughout the rehabilitation and afterwards [3, 6]. This is concerning, because prolonged sedentary behaviour is associated with an increased risk of various non-communicable diseases and all-cause mortality, even when older adults meet the recommended PA guidelines [7, 8]. Additionally, prolonged sedentary behaviour may delay functional recovery, exacerbate deconditioning, and increase dependency in activities of daily living [9]. Both excessive total sedentary time and prolonged, uninterrupted sedentary bouts contribute to these health risks [10]. Increasing the number of breaks in sedentary time and decreasing total sedentary time may be a more feasible starting point for vulnerable groups than increasing physical activity [11]. The lower physical demand might be a gateway to more activity. Nevertheless, the optimal approach to reducing sedentary behaviour in geriatric rehabilitation remains unclear.

*Monitoring and feedback* are behaviour change techniques that may help older individuals reduce and change their sedentary behaviour [12–15]. *Monitoring* involves observing a person's behaviour, and *feedback* entails receiving an evaluative response based on the observed behaviour [15]. Feedback and monitoring can facilitate behaviour change by providing insight into one’s current behaviour compared to the norm or into oneself, supporting goal setting, enhancing motivation, and strengthening self-efficacy [16]. Wearables that measure physical activity, such as the Hipper, can support monitoring and provide feedback [17, 18]. In the Netherlands the Hipper is already being used in geriatric rehabilitation, offering insight into daily physical activity. When combined with therapeutic coaching, it has demonstrated positive effects on perceived daily functioning [17]. However, the potential in monitoring and adapting sedentary behaviour in geriatric rehabilitation is unclear.

For eHealth to be successful, both usability and feasibility are essential. This may be particularly important for the complex geriatric rehabilitation context, wherein these aspects can be influenced by the interdisciplinary setting and by patients facing age-related challenges such as frailty, cognitive limitations, and fatigue [1, 19]. Usability refers to the degree to which technology can be used by intended users to achieve goals with effectiveness, efficiency, and satisfaction [20, 21]. Feasibility refers to the extent in which digital health system functions as intended [21]. Although patients and healthcare professionals perceive wearables as having the potential to influence activity behaviour, evidence regarding their usability and feasibility in geriatric rehabilitation remains limited [22–24]. Exploring healthcare professionals' perspectives on additional intervention components may further provide insight into perceived gaps in current wearable use.

Therefore, the primary aim of this study is to explore the usability and feasibility of a sensor-based monitoring system that provides feedback on sedentary behaviour during both inpatient and home-based geriatric rehabilitation, from the perspectives of patients and healthcare professionals. To provide more context, the secondary aims are to gain insight into patients’ activity patterns during rehabilitation, and to explore care professionals' preferences regarding the content of a multicomponent intervention to reduce sedentary behaviour.

## Methods

### Study design and participants

**Fig. 1:**
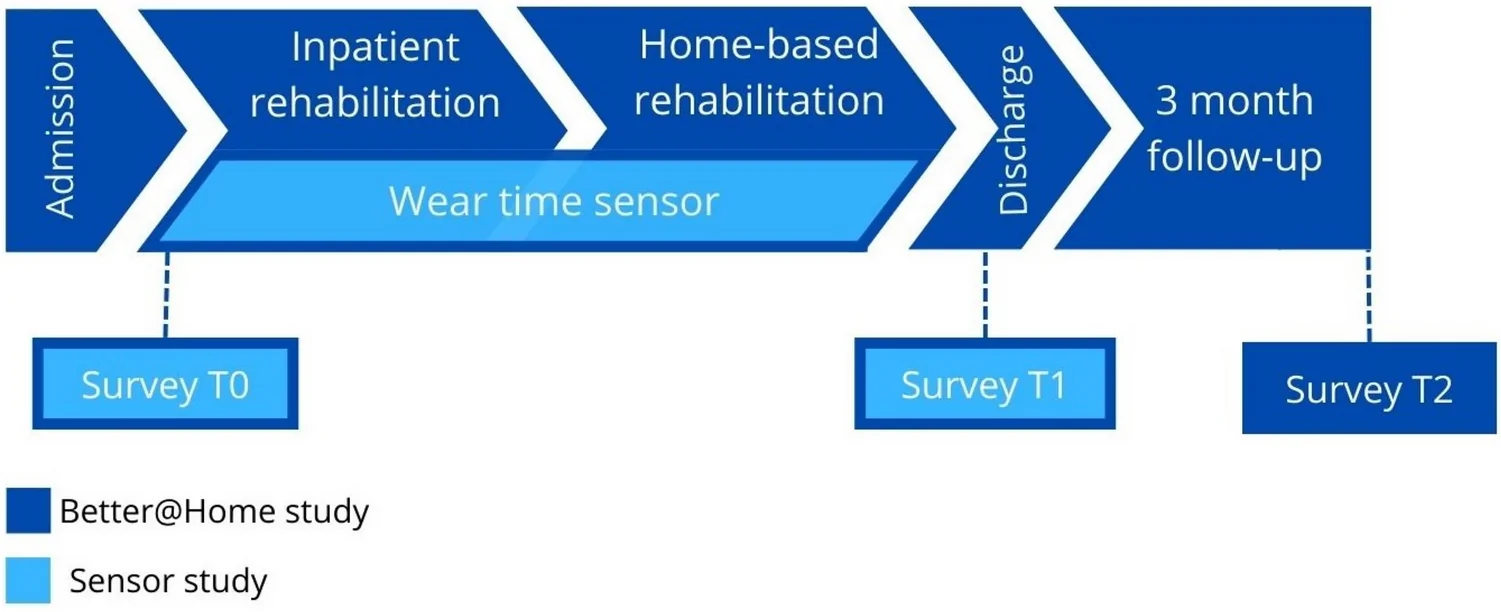
Patient journey in the Better@Home study and in this sensor study

This study is embedded in a multicentre cohort study, named Better@Home (see Fig. 1), which included 110 patients across eight geriatric rehabilitation centres [25]. Its main objective study was to “*assess the outcomes, costs and feasibility of home-based GR after shorter inpatient rehabilitation*” [25]. Participants were included during inpatient rehabilitation, with a home-based follow-up. The current mixed-methods sensor study was conducted at one of the geriatric rehabilitation centres participating in Better@Home, and patients followed their treatment programme.

All participants for this sensor study were recruited from a geriatric rehabilitation centre located in an urban area in the Netherlands offering inpatient and home-based rehabilitation. Patients and their care professionals were invited to participate. In line with the sample size of Better@Home, this study aimed to include a at least 15 patients [25]. This sample was considered sufficient to gain meaningful insights into usability and feasibility, as long as the sample adequately reflected the heterogeneity of the geriatric rehabilitation population. Patients were eligible to participate if they: (1) were admitted to the rehabilitation centre between December 2023 and November 2024; (2) were considered eligible for participation in the Better@Home program study as assessed by each facility’s multidisciplinary team based on patients' physical, cognitive, and psychological functioning; (3) lived in the community; (4) were able to speak and understand Dutch; (5) were able to provide informed consent (see the Better@Home protocol for a more detailed description) [25]. The multidisciplinary team determined whether the participant was eligible for using the sensor monitoring system. Care professionals were eligible for participation if they (1) participated in the Better@Home study, and (2) were involved in the treatment of the patients who used the sensor during the data collection. These professionals included occupational therapists and physical therapists.

The Medical Ethics Committee of University Hospital Maastricht/Maastricht University determined that the study was not subject to the Dutch Medical Research Involving Human Subjects Act (WMO) (reference number 2023–3744).

### Sensor monitoring system

The Hipper sensor monitoring system (Hipper Therapeutics B.V.) was used to assess patients' movement behaviour [18]. The system consists of multiple components: a wearable sensor (PAM AM400/AM300, 3-dimensional accelerometer sampling at 12.5Hz) that provides output every minute, a wireless transmitter (Raspberry Pi with a Z-wave shield Model B+ quadcore CPU, 1024 MB RAM), and a web-page dashboards for patients and for care professionals. The sensor measures body movement expressed by the Physical Activity Measure (PAM), which reflects the amount of movement recorded. The sensor is worn on the hip and collects data that is transferred wirelessly to the dashboards for care professionals and patients. Only the dashboards require an internet connection to function. As part of the monitoring system, care professionals coach patients on their movement behaviour based on the data collected by the sensor [17]. There was no fixed format for coaching conversations; professional could provide coaching during regular therapy sessions. The wearable sensor itself will hereinafter be referred to as "sensor", the sensor monitoring system as a whole (including the coaching by care professionals) as “sensor system”.

Three different sensors were used during this study: type 1, type 2.1, and the revised version, type 2.2. A previous version of the sensor system (with sensor type 1) was already implemented in the rehabilitation centre. Before the start of our study, type 1 was adapted into a new prototype (type 2.1). At the start of our study, we used the type 2.1 sensor, which provided an output per minute instead of 15 min as type 1 did. The increased output led to the redesign of the professional and patient dashboards, to which both patients and professionals could have access. The data were presented on the dashboard as PAM scores per minute (type 2.1 and 2.2) or per 15 min (type 1). The sensor did not provide on-device feedback. During a required update to its algorithm, we used the type 1 version. After the update we switched to type 2.2. Although the versions differed in data output and dashboard presentation, the same coaching approach was used across all versions.

### Procedure

Before the start of data collection, all care professionals involved with the sensor system underwent a refresher training on its utilization, which included coaching and a presentation by a researcher (SD) and the company providing the sensor system. Additionally, the training aimed to increase care professionals’ knowledge about the risks of sedentary behaviour and explained the differences between the previously used sensor (type 1) and the sensor to be used in the study (type 2.1).

Data collection took place between January 2024 and January 2025. Patients who participated in this study followed their treatment programme as determined by the rehabilitation team. Further details regarding the content of the rehabilitation programme can be found in the Better@Home protocol [25]. Care professionals approached patients eligible for Better@Home within 2 weeks of admittance to rehabilitation and informed them about the study with a verbal explanation and an information letter. After signing the informed consent form, all patients received a sensor system, an explanation regarding the reason for wearing the sensor, and an explanation of its application. All patients wore a sensor unless there was a valid reason, as determined by the patient or professional, not to use one. Which sensor the patient received was dependent of the date of inclusion. The patients were advised to clip the sensor to their belt while dressing in the morning and were informed that they were not required to wear the sensor at night nor during showering, bathing, or swimming. Patients wore the sensor during both inpatient and home-based rehabilitation.

The project team of the participating geriatric rehabilitation centre gathered every 6 weeks to discuss the sensor system and its application, so any arising issues could be addressed quickly.

### Data collection

We used three types of data collection. The first was a focus group with care professionals towards the end of the data collection to assess their perspective on the usability and feasibility of the intervention, as well as their preferences for a multicomponent intervention. Questions regarding a multicomponent intervention were included to inform further development and implementation of the sensor system, identify additional components considered important by professionals, and possibly provide extra insight into usability or feasibility of the current intervention. The second type of data collected were interviewer-administered surveys among patients at admission (T0) and after discharge (T1) from rehabilitation to assess their perspective on usability and feasibility of the sensor system. Thirdly, quantitative data was derived from the sensor (PAM scores ) to obtain insight into patients’ activity patterns.

### Usability and feasibility from a care professional perspective

Two researchers (one female, one male (SD, MZ)) guided the focus group. Both researchers had experience conducting focus groups and had met some of the participants prior to the focus group. The User Experience Honeycomb model was used to formulate the focus groups questions [26, 27]. The focus group was divided into small group discussions and a plenary discussion. For the small group discussions, participants were divided into two groups to discuss and respond to four statements that were written on posters: (1) *What keywords come to mind when you think about your experience with the sensor system;* (2) *I would use the sensor system more often if…;* (3) *What are the benefits of the sensor system in its current version for (a) you and (b) patients;* (4) *If an intervention is to be implemented to reduce sedentary behaviour in geriatric rehabilitation, the following must be considered…*

In the plenary discussion follow-up questions, relating to usability and to feasibility, were posed: *‘Is the current version of the sensor system useful for you as a care professional?’* and ‘*Is using the current version of the sensor system feasible for you as a care professional?’* Please refer to supplementary material 1 for the topic list. The interviewer made notes during the focus group. The plenary discussion was audio-recorded, and the recordings and digitized posters were stored in a secure and restricted data folder.

### Usability and feasibility from a patient perspective

Two interviewer-administered surveys were used for each patient: one at the start of the rehabilitation (T0) and one at the end (T1) (see Fig. 1.). At T0, the patient's characteristics were collected, and the Barthel Index was administered. No usability or feasibility questions were asked at T0. The T1 survey consisted primarily of closed-ended questions related to feasibility and usability based on the User Experience Honeycomb [26, 27]. An example question regarding feasibility is ‘*Have you received adequate support from your care professionals in using the sensor system?’.* One of the usability questions was ‘*To what extent did using the sensor system have added value for you?’.* Scoring options differed per answer. See supplementary material 2 for a list of questions and answer options related to the sensor system and its application. Reponses were recorded in Castor EDC. In addition, to assess adherence as part of feasibility, sensor wear days were used.

### Sensor data

To provide contextual information, activity patterns, recorded as minute-by-minute activity data in a PAM-score, were retrieved from the sensor worn by the patients. Higher PAM scores indicate higher levels of activity. The retrieved data was summarized as described in Box 1. To convert PAM scores to movement variables, scores were converted to MET scores (MET= (PAM/100) +1). For the data to be included in the analysis, the sensor had to be worn for a period of at least 4 days. This applies to both the inpatient phase and the home-based phase. The activity data from the sensor system was obtained as a PAM score per minute or per 15 min. Due to the limited number of participants per sensor version, we did not stratify the analysis by sensor version.

**Box 1:** Types of movement variables

Variable	Description
Sedentary time	Waking time (with a minimal duration of 1 min) spent on activities characterized by an energy expenditure below or equal to 1.5 MET [5]
Light physical activity (LPA)	Time (with a minimal duration of one minute) spent on light physical activity characterized by an energy expenditure between 1.5 MET and 3.0 MET
Moderate to vigorous physical activity (MVPA)	Time (with a minimal duration of one minute) spent on moderate to vigorous physical activities characterized by an energy expenditure above or equal to 3.0 MET
Prolonged sedentary bout duration	Time spent in an uninterrupted period of sedentary behaviour [28], with a minimal length of 30 min
Half-life bout duration (W50%)	A weighted median bout duration in which the sedentary bout duration above and below half of all sedentary time is accumulated [29, 30]

### Analysis

Qualitative and quantitative findings were analysed and presented separately. They were integrated during the interpretation phase by comparing findings of both methods to identify similarities and differences.

### Usability and feasibility from a care professional perspective

The focus group’s transcript was coded and analysed according to the Framework method principles [31]. The analysis was used to identify key elements and common themes regarding usability, feasibility, and preferences for a multicomponent intervention. Following the Framework method, the audio recording was first transcribed (by SD) and pseudonymized. Transcripts were not provided to participants. SD then familiarized herself with the data by checking the transcripts. Two researchers (SD, MH) performed the initial inductive coding of the transcript. SD and MH then compared and discussed their coding in a consensus meeting. Discrepancies were resolved through discussion, and the codebook was refined accordingly. SD applied the refined codebook to the focus group and notes on posters in Atlas.ti 23. The data was summarized in a matrix and illustrated with quotations. Finally, the data was interpreted, and the findings were discussed with the rehabilitation centre project team.

### Usability and feasibility from a patient perspective

Descriptive statistics were used to examine the characteristics of study participants and to analyse survey data responses. Data was not normally distributed and was therefore summarized as medians and interquartile ranges. Categorical data is presented as percentages. Open-ended responses were grouped and summarized.

### Sensor data

Data from all sensor versions were analysed. Only data marked as *awake (*defined as data collected between the first and last registered movement during a day) and from days marked as *valid* (PAM day score >1) were included in the analysis. Data from the first and last days of measurement were excluded due to incompleteness. Adherence was calculated by dividing the number of days with a PAM day score exceeding 1, by the total number of days recorded by the sensor system. The movement variables (Box 1) were averaged per person and stratified by rehabilitation phase (inpatient vs. home based). Data was pooled, and outcomes were analysed descriptively, reported as medians and interquartile ranges (IQR). Statistical analyses were performed using IBM SPSS statistics version 29.

## Results

### Baseline characteristics

**Fig. 2:**
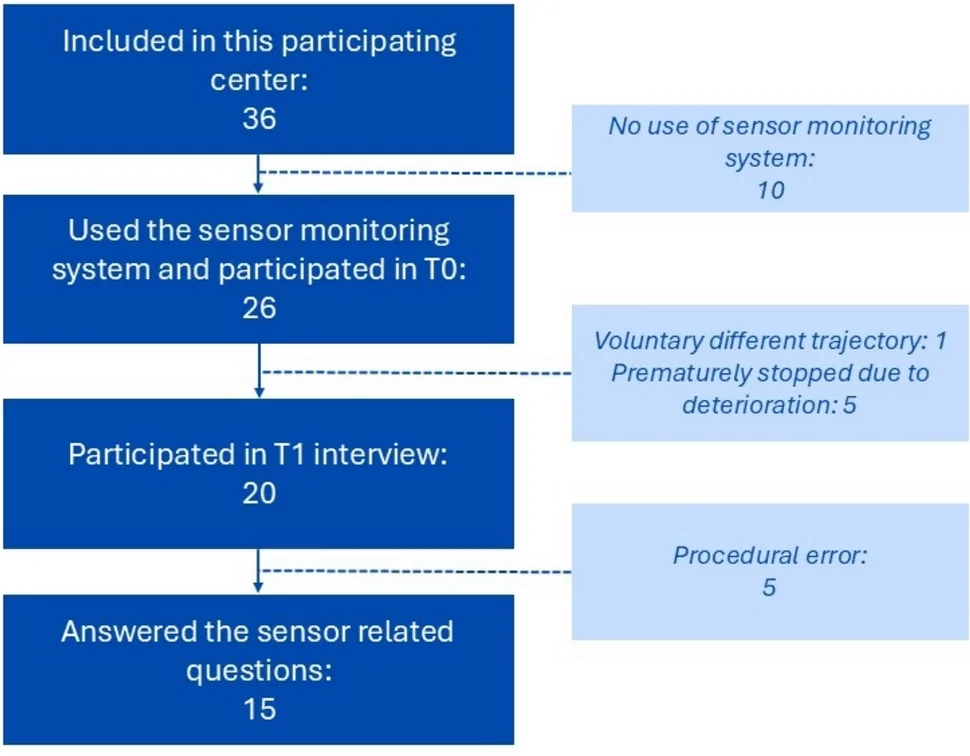
Flowchart showing the number of participants during the study

The Better@Home study included 36 patients from the participating geriatric rehabilitation centre. The multidisciplinary team determined that eight patients were not eligible to use the sensor system. For two patients no sensor system was available. So, in this sensor study, 26 patients were included. Twenty patients participated in the T1 interviewer-administered survey (see Fig. 2). Due to a procedural error during data collection, only 15 patients answered the sensor-related survey questions.

The median age of the patients was 77 years (IQR 70.5–84.3), 16 patients were female (61.5%), and 18 patients lived independently and alone (69.2%). Almost 35% of patients were in the rehabilitation centre due to elective orthopedy/trauma. The second most common diagnosis was respiratory disease (23.1%). Patients spent a median of 25.5 days (IQR 19.0–45.0) in inpatient rehabilitation and 46.5 days (IQR 34.0–78.0) at home during their treatment. Further details are included in Table 1. Baseline characteristics were similar between patients who answered the sensor-related questions and those who did not. Of the nine participating care professionals, seven (77.8%) were physical therapists and two were occupational therapists. Seven of the participating care professionals (77.8%) were female.

**Table 1:** Characteristics of participating patients (*N* = 26)

Median (IQR) age in years	77 (70.5–84.3)
Female (*N* (%))	16 (61.5)
Living situation (*N* (%))	
Independent, alone	18 (69.2)
Independent, with other persons	8 (30.8)
Diagnosis (N, (%))	
Elective orthopedy/trauma	9 (34.6)
Respiratory diseases	6 (23.1)
Stroke	5 (19.2)
Oncological diseases	2 (7.7)
Amputations	1 (3.8)
Falling and kidney insufficiency	1 (3.8)
Intestinal surgery	1 (3.8)
Rib fracture	1 (3.8)
Barthel Index (N, (%))	
Total dependency	4 (15.4)
Severe dependency	4 (15.4)
Moderate dependency	13 (50.0)
Slight dependency	5 (19.2)
Median length of stay in days (IQR)	
Inpatient (*n* = 26)	25.5 (19.0–40.5)
Home based (*n* = 24)	46.5 (34.0–78.0)
Inpatient + home based (*n* = 24)	84.5 (57.3–107.3)

### Usability and feasibility from a care professional perspective

Three major themes emerged from the focus group regarding the primary objective, i.e. the purpose of using the sensor system, experiences with using the sensor system, and consequences of the experiences. For the secondary objective regarding preferences for a multicomponent intervention, the following themes emerged: the preferences, roles in using the sensor system, and the importance of the rehabilitation climate. An overview of themes and illustrative quotes is provided in supplementary material 3.

Regarding the application of the sensor system, care professionals indicated that the purpose of using the sensor system was to discuss and map a patient's load and capacity balance. They considered its use to be successful only when it provided a patient insight into their own activity and when patients changed their behaviour accordingly, especially when they needed extra support to change their activity. The care professionals found the sensor system to be a useful conversation starter, enabling meaningful and motivational conversations. They indicated that it provided insight into patients’ activity patterns and intensity, both for them and their patients. As one professional said:

“*You can see, at least with the old sensor [type 1], when someone has moved. And the intensity of that movement. And you can also see if someone practices all morning and does nothing in the afternoon—which is what you don't want—or when someone becomes inactive after six o'clock.” (D1, physical therapist).*

On the other hand, professionals noted a number of usability issues, including the PAM score as a source of unclarity. As one of them stated:

*"The PAM [physical activity measure] score is unclear; a patient doesn't understand what 3, 4 or 20 means." (D3, occupational therapist)*

Other experiences that substantially decreased the usability of the sensor system were using it without a clear patient treatment goal and the perceived inaccuracy of the measurements. Despite the sensor being updated during the study, care professionals still felt the usability was insufficient. They felt like the data from the sensor system had low credibility. One provided a clear example:

*"I actually had a patient where I said ‘Ah, you didn’t take the stairs, because look, it only shows this…’ She was really offended, because she had been working very hard… the sensor system just hadn't recorded anything." (D7, physiotherapist).*

Furthermore, due to the increase in datapoints, the graphs belonging to sensor types 2.1 and 2.2 were perceived as more complex and less interpretable than those from sensor type 1. This resulted in feelings of frustration among some professionals. The care professionals considered the dashboard to be accessible for patients with basic digital skills, but they also thought that patients often do not have these skills. The different steps necessary to access the dashboard also reduced its feasibility*.* Professionals furthermore felt that technology like this sensor system will be important in future rehabilitation. However, the feasibility of the sensor system was also reduced by practical complications, including the risk of device loss, logistical issues, and the number of steps required to include a patient in the system and transfer the patient to the home-based setting.

### Usability and feasibility from a patient perspective

Fifteen patients answered the survey questions regarding the usability and feasibility of the sensor system. Twelve of them stated that their care professional asked them if they wanted to use the sensor system. One patient reported that the question was not asked, and two could not recall whether it was asked. Thirteen patients reported using the sensor system during both the inpatient and home-based rehabilitation, indicating a high level of adherence. However, the use and usability of the sensor system were not clear to all users. Four patients thought the purpose of the sensor system was to change their level or awareness of activity. Three other patients thought the purpose was so the care professionals could monitor their behaviour. In addition, responses on why it was used included: "*for research"*, "*I didn't think about that"* and *‘"I don't know, I'm in a wheelchair*". Regarding the trustworthiness of the sensor, seven patients said they (partially) trusted it, while the others did not.

Eight patients, slightly more than half, reported the sensor system was easy to use. Thirteen patients indicated that using the sensor system offered no added value or advantages. One reported it had some value, and another noted that the sensor facilitated external accountability for exercise adherence. Seven participants did not experience any disadvantages from using the sensor system. Among the patients who did, one in five indicated that the sensor kept coming off. Other disadvantages included forgetting the sensor, easily losing it, or breaking it. Patients did not mention the dashboard. Despite the disadvantages, five patients said that they would recommend the sensor system, and four were undecided. The remaining six patients would not recommend it.

Regarding the support patients experienced with using the sensor system, almost half, i.e. seven patients, reported that the care professionals did not provide adequate support. Three patients indicated that they would have preferred to review the results together with the care professional during therapy, and two expressed a desire for more information about the sensor and its use. Only one patient was guided by an informal caregiver in using the sensor system.

### Care professionals’ preferences for a multicomponent intervention

When the care professionals were asked about the desired components of an intervention to decrease sedentary behaviour, they emphasized that an intervention should be easy to use, have a clear objective, and should incorporate technology. They highlighted the importance of increasing patients’ and informal caregivers’ knowledge about sedentary behaviour, emphasizing that the technology should motivate patients to engage in physical activity. Furthermore, they wanted a pre-implementation analysis of the technology to be used (e.g. what do we need, how do we want to use it, and which technology is available). Ideally, the technology should provide direct visual or tactile feedback and offer insight into patterns, intensity, and type of activity. They noted:

*"… insight into when someone is active and for how long (D3, occupational therapist)*

*… and the type of activity, that would be good too (D6, physiotherapist)*

*… and at what time during the day (D9, physiotherapist)*

*… and the development over time." (D2, physiotherapist)*

Care professionals identified the general rehabilitation climate as highly important; the culture should invite being physically active, be safe to move around in, and care professionals should act as role models. They also described several preconditions for a multicomponent intervention. First and foremost, the entire rehabilitation team should be involved in the intervention.

### Sensor data

Sensor data from the 26 patients who used a sensor showed that 16 participants used it during both inpatient and home-based rehabilitation. Twenty-three patients used the sensor during inpatient rehabilitation. These 23 patients had a median use of 11.0 days (IQR 7.0–22.0), which corresponds to a median use of 100% (IQR 73.3–100.0) of the days the sensor was offered. During home-based rehabilitation, 19 patients used the sensor. The sensor was offered to them for a longer period (median: 34.0 days [IQR 25.0–45.0]). Patients used the sensor on 96.6% (94.1–100.0) of those days.

### Activity pattern and intensity

During the inpatient rehabilitation, patients spent an average of 93.0% (IQR 81.2–97.3), or 665.8 (IQR 599.5–743.6) minutes of their waking day in sedentary behaviour. During home-based rehabilitation, this was 90.5% (IQR 79.9–97.7). However, as total daily wear time increased in the home setting, the average absolute time spent in sedentary behaviour increased to 704.2 (IQR 600.4–896.4) minutes during home-based rehabilitation. The percentage of time spent engaged in MVPA was in both periods 0.0 (IQR 0.0–0.2). The percentage of time spent engaged in  light physical activity was 6.9% (IQR 2.4–18.7) in the inpatient setting and increased by 14.1 min (2.6%) in the home-based setting.

The pattern and duration of sedentary bouts also showed differences between the inpatient period and the home-based period. The number of bouts was 37.9 (IQR 20.4–58.6) inpatient and 55.5 (IQR 37.4–78.8) home based. The median bout length inpatient was 22.7 (IQR 10.0–34.3) minutes and home based 14.9 (IQR 9.4–20.3) minutes. The time spent in bouts longer than 60 min decreased by 47.9 min (10.6%). More detailed outcomes are presented in Table 2 and supplementary material 4.

**Table 2:** Activity pattern and intensity during rehabilitation

	Inpatient *N *= *23*	Home based *N *= *19*
Movement variables	Median (IQR) per day	Median (IQR) per day
Minutes wear time	730.0 (648.8—844.8)	796.6 (707.8—959.0)
% spent in SB	93.0 (81.2—97.3)	90.5 (79.9—97.7)
% spent in LPA	6.9 (2.4—18.7)	9.5 (2.3—19.8)
% spent in MVPA	0.0 (0.0—0.2)	0.0 (0.0—0.2)
PAM score	5.8 (3.7—11.0)	8.8 (5.3–14.9)

Sedentary bouts*	Inpatient *N*=*19*	Home based *N*=*14*
Number of bouts	37.9 (20.4–58.6)	55.5 (37.5–78.8)
% spent in bouts 31–60 min	16.2 (10.6–20.3)	17.7 (11.4–21.9)
% spent in bouts >60 min	42.4 (29.2–67.1)	31.8 (18.7–40.4)
Bout length in minutes	22.7 (10.0–34.3)	14.9 (9.4–20.3)
half-life bout duration	24.0 (6.0—77.0)	30.0 (19.0–46.3)

*Bout outcomes are derived only from sensors that measured every minute, while some patients wore sensors that measured every 15 min. This is why the sample size for these outcomes is slightly smaller. SB = sedentary behaviour, LPA = light physical activity, MVPA = moderate to vigorous physical activity

## Discussion

This study explored the perspectives of care professionals and patients on the usability and feasibility of a sensor system tracking sedentary behaviour during inpatient and home-based geriatric rehabilitation. The system was found to be easy to use, support motivational conversations, and provide insight into patients’ activity levels and patterns. Yet its overall perceived usability was limited due to concerns regarding measurement accuracy, a lack of clear goals for patients, and a lack of added value to the rehabilitation process perceived by both patients and professionals. While high adherence indicates promising feasibility, challenges such as experiences of limited support and logistical constraints reduce the feasibility level to reasonable. Patients seemed to be sedentary more than 90% of their time, with light physical activity recorded between 7 and 10% of the time. These outcomes should be interpreted with caution due to small sample size and various sensor versions. Care professionals emphasized that a multicomponent intervention to decrease sedentary behaviour should be easy to use, include technology that provides feedback and motivates patients, involve the whole team, and offer a challenging rehabilitation environment.

Participants described limited usability of the sensor system. At the same time, care professionals did describe the sensor system as a useful motivational conversation starter that provided insight into a patient’s activity pattern. The prominent issues that emerged on both the technology (e.g. measurement accuracy) and implementation level (e.g. tailored use and added value) correspond with existing literature [32–35]. Measurement inaccuracies may contribute to user fatigue and discourage sustained use, which may have been experienced by the professionals using the three sensor types [36]. The usability issues reported by patients may also be related to the geriatric specific challenges including cognitive, motivational, perceptual or physical limitations [19, 37]. The perceived added value was not high for most participants. This perceived value is also called performance expectancy and is one of the key predictors for professionals and older individuals accepting an eHealth intervention [32, 35, 38]. Some studies even suggest that high performance expectancy can overrule other implementation challenges [32]. Given the usability concerns raised, the added value of sensor monitoring and feedback technology needs to become more apparent to patients and care professionals. Strategies to do so may include educating professionals or training eHealth advocates to support successful implementation of usable eHealth [32].

Furthermore, our findings on feasibility are consistent with a systematic review on the use of eHealth in geriatric rehabilitation that stated that eHealth interventions in this setting show high adherence and can be feasible [39]. However, it must be acknowledged that adherence may partly reflect compliance with clinician instructions rather than engagement with the sensor system itself. Feasibility was challenged by both technological issues as implementation issues such as inadequate support. To ensure a system is used as originally intended, it is essential to provide adequate assistance and training for both patients and care professionals [23, 24, 33, 40, 41]. This support is particularly important in populations that have more problems with accessing, using, and understanding eHealth, such as older adults [41]. By providing adequate support, patients may also better understand the purpose and added value of the sensor system. The support should be aligned with the treatment programme and consist of both technological assistance and support on the use and interpretation of the system for both care professionals and patients [24].

The sedentary time and light physical activity of patients in this study collected by the sensor, fall within the expected range when compared to other older patient populations (although definitions in literature vary) [3, 42–45]. Our findings should, however, be interpreted with caution as sample size was small and the amount of self-monitoring and feedback was not quantified but may have influenced activity behaviour. During inpatient treatment, sedentary time of patients can range from 650 to over a 1000 min per day [42, 45, 46]. Studies show that this high level of sedentary time remains relatively stable throughout the inpatient rehabilitation [3, 46]. This is concerning, as reduced sedentary time and more frequent breaks are associated with lower levels of physical dependency and higher functional recovery [42, 47]. On the other hand, other studies also indicate that the sedentary time of patients following the transition to a home-based care programme seems to decrease naturally, by up to 45 min [45, 48]. This may explain the observed relative decrease in sedentary time between the two settings in the present study.

Exploring preferences for a multicomponent intervention also provided insight into how the sensor system could be better integrated into treatment programmes. The care professionals stated that they would prefer an intervention with a clearly defined objective that utilizes technology such as sensors. As noted in the scoping review by Petrusevski et al. (2021), such interventions can be feasible for community-dwelling older adults and have the potential to reduce sedentary time by 24–130 min per day [49]. This can be a good starting point for the geriatric rehabilitation setting as well. Successful interventions included educational components and frequently incorporated self-monitoring and health coaching [49]. These are also components the professionals in this study would like to see in a future intervention. Also, the professionals' wish for a pre-implementation analysis aligns with literature that emphasizes the importance of assessing: 1) the current situation, including existing structures, processes, challenges, and needs; 2) values associated with the intervention; and 3) stakeholders’ perspectives. These assessments are necessary to overcome usability and feasibility issues on both technological end implementation level [41, 50]. Moreover, the literature emphasizes that successful integration requires incorporating the role of the users into the organizational structure and processes, ensuring that technology aligns with these structures and processes, and deploying professionals in ways that support the intended outcomes of care [51]. Lastly, the need for a physically and culturally challenging rehabilitation environment to optimize outcomes, as indicated by professionals in our study, aligns with literature on this topic [52]. Taken together and supported by a broad analysis upfront, these different components could lead to improved usability and feasibility for care professionals.

### Strengths and limitations

The different research methods used in this study provided broad insight into the usability and feasibility of the sensor system, highlighting its notable strength. Another strength is that we included the perspective of end-users of the sensor system in both inpatient and home-based settings.

The study also had some limitations. First, the different versions of the sensor used during the study may have affected qualitative and quantitative outcomes, resulted in the absence of minute data for some patients, and had not been formally evaluated. Nevertheless, the results still provide context on patients’ activity levels and patterns during inpatient and home-based geriatric rehabilitation. Second, the usability and feasibility questions were not validated. Consequently, the findings should be interpreted with caution, and comparability with results from other studies is limited. Furthermore, not all included patients were interviewed at T1. Nevertheless, the collected responses provide valuable insights into the topic. Additionally, the results raised questions about whether coaching was carried out properly for all patients. Finally, the study was conducted in a selected sample in one rehabilitation centre, with only physical therapists and occupational therapists participating in the focus group. This may reduce the generalizability of the results.

## Conclusion

This study demonstrated reasonable feasibility and limited usability of a sensor monitoring system that provides feedback on sedentary behaviour in a geriatric rehabilitation setting. The primary concerns included measurement accuracy, a lack of clear goals for the patient, the lack of added value, and practical considerations. On the other hand, a sensor monitoring system in geriatric rehabilitation can be feasible and a valuable tool to facilitate motivational conversations. Participants emphasized the need for an implementation analysis to ensure the contextual fit and usability of a sensor monitoring system in rehabilitation. They also highlighted the need for a user-friendly system that is, accurate, aligned with patient goals, and supported by the entire, multidisciplinary team. Furthermore, user support is required for both care professionals and patients. These findings underline the importance of designing eHealth for geriatric rehabilitation that is not only functional, but also responsive to the needs of both patients and professionals, thereby ensuring that reducing sedentary behaviour becomes a shared and achievable goal.

## Supplementary Information

Below is the link to the electronic supplementary material.

Supplementary file1 (PDF 391 KB)

Supplementary file2 (PDF 320 KB)

Supplementary file3 (PDF 249 KB)

Supplementary file4 (PDF 394 KB)

## Data Availability

The datasets generated during and/or analyzed during the current study are available from the corresponding author on reasonable request.
