# Supplementary material for: Usability and feasibility of a wearable sensor-based monitoring system providing feedback on sedentary behaviour during inpatient and home-based geriatric rehabilitation
Source: Eur Geriatr Med. 2026 Oct 6;17(5):2955–66. doi: 10.1007/s41999-026-01616-6 (PMC13755094; doi:10.1007/s41999-026-01616-6)
Supplement: Supplementary file 1 [file 41999_2026_1616_MOESM1_ESM.pdf]

Digital Geriatric Medicine, special issue of European Geriatric Medicine

Usability and feasibility of a wearable sensor providing feedback on sedentary behavior during inpatient and home-based geriatric rehabilitation

Suzanne M. Debeij, MSc; Miriam L. Haaksma, PhD; Margot W.M. de Waal, PhD; Jolanda C.M. van Haastregt, PhD; Margriet C. Pol, PhD; Marise J Kasteleyn, PhD; Eléonore F. van Dam van Isselt, MD PhD

Corresponding Author
Suzanne M. Debeij, MSc
Department of Public Health and Primary Care
Leiden University Medical Center
Leiden
The Netherlands
Phone: +31 6 513 06 514
Email: s.m.debeij@lumc.nl

Supplementary material 1. Topic list focus group

Roughly translated from Dutch to English

Introduction

"Good afternoon, very nice that you are all available today to talk to each other about the use of the sensor. For those who don't know me, my name is SD and I am a PhD candidate. I don't work for the company of the sensor monitoring system. I am researching the use of eHealth in geriatric rehabilitation and will lead the focus group today. There is MD, who is the second in this focus group today.

In the past year you have used the sensor monitoring system. The difference with the years before was that it was used with more patients, and the sensor monitoring system itself was a little different than before. Today I am curious about your experience with this and your ideas for improvement. We'll start with a round of introductions. Then you will split up into groups to different posters. Each poster contains a question or a statement. It is up to you to write down your opinion about this on the posters. After about 5 minutes I indicate that there is still some time to finish. When the time is up and you go with your group to the next poster, until you have been to all of them. Then we all come together to discuss together what has come forward and why it has come forward.
The presentation and discussion of the posters together will be recorded. In the analysis, your data is anonymized, and the material is not shared outside the research team, so you are completely anonymous in the analysis.

I will start the recording in a moment and ask one of your representatives if it is okay if the conversation is recorded. Are there people who don't like it?"

- Start recording-

"Do you agree that the conversation is recorded?"

Introduction (5-10 minutes)

I would like to ask you all to introduce yourselves. Would you like to indicate the following?

- Name
- Role within the organization
- Familiarity with the sensor monitoring system prior to the study

Thank you. Now let's move on to the posters. You can divide into groups at the posters, make sure the groups are approximately the same size. It doesn't matter which poster you start with, you will get to all of them.

Core (± 7 minutes per poster (based on 4 posters)) (±40 minutes total)

Poster 1: What keywords come to mind when you think about your experience with the sensor system?

Any further questions for discussion:

a. Why?
b. Is the information within the dashboard easy to find?
c. What do you think of the simplicity of use?
    i. Is it accessible to patients?

Poster 2: I would use the sensor monitoring system more often if...

Any further questions for discussion:

d. Is it feasible for you as a professional to use the current version of the sensor monitoring system?
    i. And for patients?
e. Statement: I (sometimes) prefer not to use the sensor monitoring system because..? (What are barriers?)
    i. What prevents you from using the sensor?
    ii. Why not use the sensor?
    iii. What are the impeding factors and the facilitating factors for the deployment of the sensor?
f. Can the sensor be trusted and believed?

Poster 3: What are the benefits of the sensor system in its current version for (a) you and (b) patients?

Any further questions for discussion:

a. When is it successful for you?
b. What would you like to get out of the use of the sensor?/ What would you like to use the sensor for?
    i. *Why would you use the sensor, what could the sensor do for patients, informal caregivers and professionals?*
    ii. Is the current version of sensor useful for you as a professional?
        1. And for patients?
c. What are the main advantages of using the sensor?
    iii. What are the impeding factors and the facilitating factors for the deployment of the sensor?

Poster 4: If an intervention is to be implemented to reduce sedentary behavior in geriatric rehabilitation, the following must be considered…

a. What kind of aspects should an intervention consist of?
b. Who are important to include in a broad intervention?
    iv. Who will work with it in practice?

c. Does the sensor fit these needs and interests? Why?

Closing (5-10 minutes)

"Thank you for your input We have arrived at the conclusion of the focus group. I have some final questions."

- What do you think are the most important points we discussed today?
- Is there anything else that you feel has not been discussed and that could be important for the research?
- Would you like to share something else?

"If you think of something later that you want to share, you can contact me by email ([mail]) or telephone ([phone number]). We would like to thank you very much for your participation in this focus group."

"Have a nice evening"
