# Supplementary material for: Usability and feasibility of a wearable sensor-based monitoring system providing feedback on sedentary behaviour during inpatient and home-based geriatric rehabilitation
Source: Eur Geriatr Med. 2026 Oct 6;17(5):2955–66. doi: 10.1007/s41999-026-01616-6 (PMC13755094; doi:10.1007/s41999-026-01616-6)
Supplement: Supplementary file 2 [file 41999_2026_1616_MOESM2_ESM.pdf]

*Digital Geriatric Medicine, special issue of European Geriatric Medicine*

**Usability and feasibility of a wearable sensor providing feedback on sedentary behavior during inpatient and home-based geriatric rehabilitation**

Suzanne M. Debeij, MSc; Miriam L. Haaksma, PhD; Margot W.M. de Waal, PhD; Jolanda C.M. van Haastregt, PhD; Margriet C. Pol, PhD; Marise J Kasteleyn, PhD; Eléonore F. van Dam van Isselt, MD PhD

**Corresponding Author**
Suzanne M. Debeij, MSc
Department of Public Health and Primary Care
Leiden University Medical Center
Leiden
The Netherlands
Phone: +31 6 513 06 514
Email: s.m.debeij@lumc.nl

## Supplementary material 2. Questions in the survey relating to the use of the sensor

| Question | Answer options* | Related to |
|---|---|---|
| *Questions about the use of eHealth* | | |
| Have your healthcare providers asked you whether you wanted to use eHealth applications during your treatment, such as video calling, rehabilitation apps, sensors, or other applications? | Yes; No; I do not know | F |
| Did you use eHealth applications such as video calling, sensors, a rehabilitation app, or other applications during your treatment? | No (why not?) > I did not want to; I cannot deal with that; HCPs did not think it was necessary; I do not know; other… (open answer);<br>Yes (which ones?) > sensors; videocalls; rehabilitation application; other… (open answer) | F |
| When did you use these eHealth applications? | During inpatient care; during home-based care | F |
| *Questions about the usability and feasibility of the sensor* | | |
| Why do you think the sensor is used during your rehabilitation? | To become more aware of my movement; to become more aware of my sitting; to change my activity pattern; so the therapist can see what I am doing; I do not know; other… (open answer) | U |
| Did you trust the information the sensor gave you? | Yes, fully; yes, a little bit; no | U |
| How easy or difficult did you find using the sensor? | I thought the use of the sensor was… difficult; not easy, not difficult; easy | U |
| To what extend did using the sensor have added value for you? | For me, the use of the sensor had… no added value; some added value; much added value<br>The added value was…. (open answer) | B |

| | | |
|---|---|---|
| What do you think are advantages of using the sensor during your rehabilitation? | (Open answer) | B |
| What do you think are disadvantages of using the sensor during your rehabilitation? | (Open answer) | B |
| Would you recommend using the sensor to other patients? | Yes of course; I am not sure; no, I would not recommend it | B |
| Have you been properly supported by your HCPs in using the sensor? | Yes, very well; yes, reasonably well; no, not well | F |
| What could have been improved in the support from the HCPs when using the sensor? | Nothing; the following… (open answer) | F |
| Did you have help from your loved ones when using the sensor? | Yes; no; not applicable | F |

**Answer options are separated by a semicolon, F=Feasibility, U=Usability, B=Both usability and feasibility*
