# Supplementary material for: Usability and feasibility of a wearable sensor-based monitoring system providing feedback on sedentary behaviour during inpatient and home-based geriatric rehabilitation
Source: Eur Geriatr Med. 2026 Oct 6;17(5):2955–66. doi: 10.1007/s41999-026-01616-6 (PMC13755094; doi:10.1007/s41999-026-01616-6)
Supplement: Supplementary file 3 [file 41999_2026_1616_MOESM3_ESM.pdf]

Digital Geriatric Medicine, special issue of European Geriatric Medicine

Usability and feasibility of a wearable sensor providing feedback on sedentary behavior during inpatient and home-based geriatric rehabilitation

Suzanne M. Debeij, MSc; Miriam L. Haaksma, PhD; Margot W.M. de Waal, PhD; Jolanda C.M. van Haastregt, PhD; Margriet C. Pol, PhD; Marise J Kasteleyn, PhD; Eléonore F. van Dam van Isselt, MD PhD

Corresponding Author
Suzanne M. Debeij, MSc
Department of Public Health and Primary Care
Leiden University Medical Center
Leiden
The Netherlands
Phone: +31 6 513 06 514
Email: s.m.debeij@lumc.nl

Supplementary material 3.

Themes	Quotes
Experience of current use – positive	• *“You can see, at least with the old sensor [type 1], when someone has moved. And the intensity of that movement. And you can also see if someone practices all morning and does nothing in the afternoon – which is what you don't want – or when someone becomes inactive after six o'clock.” (D1, physical therapist)*<br>• *Especially when you think, okay, this person needs a bit more time, or really needs additional insight to make progress... if a regular conversation, or a simpler non-digital approach, is not enough (D8, physical therapist)*
Experience of current use - negative	• *-"The PAM [physical activity measure] score is unclear; a patient doesn't understand what 3, 4 or 20 means." (D3, occupational therapist)*<br>• *It doesn’t provide enough benefit yet. It certainly helps sometimes, but not often enough (D8, physical therapist*<br>• *(about handover of dashboard and coaching between care professionals) “That means there are extra steps to do, and the more extra steps there are, the more likely these kinds of things (long time between handover) are to happen. They are small issues, I agree, but they can slip in more easily because the system is set up that way” (D2, physical therapist)*
Consequence of negative experience	• *“And that's not something the sensor can do anything about, but coincidentally, among the last three sensors I deployed, one patient became ill, one was readmitted, and one suddenly passed away. That's quite a hassle, because the sensor is suddenly gone. You end up having to call family members who are grieving to ask whether they could please return the device. That's definitely an issue. And nobody can really do anything about it, but it sometimes makes me hesitant to use it in outpatient care. I find myself thinking: if I don't give it to them, I won't have to follow up on it afterwards. (D7, physical therapist)*<br>*Yes, it literally creates extra hassle…. in various ways” (D3, occupational therapist*
Goal of use	• *“For me, the main focus is really on the balance between activity demands and capacity. That's my primary goal.” (D8, physical therapist)*<br>• *“If someone acts on the information they see.” (D1, physical therapist)*

Roles	• *[about reducing sedentary behaviour] "Yes, but sometimes I wonder whether we can really achieve that here, or whether it needs to come much more from the government. Because if it were talked about much more often on television, for example in the news, or if the government communicated more clearly about it, then... (D3, occupational therapist)*<br>*I think it needs to come from all sectors. (D8, physical therapist)*<br>*[Others agree]*<br>*That way, you can start making a difference everywhere." (D9, physical therapist)*
Rehabilitation climate	• *"I think other prerequisites need to be in place first, such as a supportive physical environment. A sensor may encourage someone to be more active, but if the environment is not safe enough or does not facilitate activity, acting on that advice can be difficult. For example, if the sensor says, 'be more active,' but there is nowhere safe to stand or the person is unable to walk, it becomes very challenging to act on that feedback." (D9, physical therapist)*
Preferences for movement intervention	• *"... insight into when someone is active and for how long (D3, occupational therapist)*<br>*... and the type of activity, that would be good too (D6, physiotherapist)*<br>*... and at what time during the day (D9, physiotherapist)*<br>*... and the development over time." (D2, physiotherapist)*<br>• *"Some form of direct visual feedback is important. Not something that requires you to sit down, open something and check it… [agreement by others], but rather, for example, when I go to the bathroom and happen to pick up the device, I immediately think, 'oh wow, I have already twenty today'. " (D7, physical therapist)*
