# Supplementary material for: Usability and feasibility of a wearable sensor-based monitoring system providing feedback on sedentary behaviour during inpatient and home-based geriatric rehabilitation
Source: Eur Geriatr Med. 2026 Oct 6;17(5):2955–66. doi: 10.1007/s41999-026-01616-6 (PMC13755094; doi:10.1007/s41999-026-01616-6)
Supplement: Supplementary file 4 [file 41999_2026_1616_MOESM4_ESM.pdf]

Digital Geriatric Medicine, special issue of European Geriatric Medicine

Usability and feasibility of a wearable sensor providing feedback on sedentary behavior during inpatient and home-based geriatric rehabilitation

Suzanne M. Debeij, MSc; Miriam L. Haaksma, PhD; Margot W.M. de Waal, PhD; Jolanda C.M. van Haastregt, PhD; Margriet C. Pol, PhD; Marise J Kasteleyn, PhD; Eléonore F. van Dam van Isselt, MD PhD

Corresponding Author
Suzanne M. Debeij, MSc
Department of Public Health and Primary Care
Leiden University Medical Center
Leiden
The Netherlands
Phone: +31 6 513 06 514
Email: s.m.debeij@lumc.nl

Supplementary material 4. Detailed outcomes of movement variables, divided per sensor version

	Inpatient data			
	**All**	**Type 1**	**Type 2.1**	**Type 2.2**
**Movement during the day** ***Median (IQR)***	***N=23***	***N=5***	***N=6***	***N=13***
Wear time	730.0 (648.8 – 844.8)	788.6 (688.8 – 870.4)	793.3 (657.3 – 1149.6)	730.0 (648.7 – 844.8)
%Spent in SB	93.0 (81.2 – 97.3)	99.5 (96.7 – 99.0)	96.5 (94.6 – 98.0)	85.7 (79.1 – 92.3)
Minutes in SB	665.8 (599.5 – 743.6)	743.6 (688.1 – 864.0)	760.8 (639.3 – 1085.5)	623.9 (579.0 – 672.2)
%Spent in LPA	6.9 (2.4 – 18.7)	0.5 (0.1 – 3.3)	3.2 (2.0 – 5.1)	14.3 (7.7 – 20.7)
Minutes in LPA	49.9 (25.5 – 126.7)	6.0 (0.6 – 25.9)	26.2 (16.3 – 43.8)	105.8 (56.1 – 164.8)
%Spent in MVPA	0.0 (0.0 – 0.2)	0.0 (0.0 – 0.0)	0.3 (0.1 – 0.3)	0.0 (0.0 – 0.1)
Mean minutes in MVPA	0.3 (0.0 – 1.0)	0.0 (0.0 – 0.0)	2.1 (0.9 – 2.8)	0.3 (0.2 – 0.4)
PAM score	5.8 (3.7 – 11.0)	4.1 (3.5 – 8.7)	3.4 (2.3 – 5.5)	10.0 (5.6 – 14.3)
**Bouts***	***N=19***	**n.a.**	***N=6***	***N=13***
Number of bouts	37.9 (20.4 – 58.6)	n.a.	19.4 (12.3 – 29.1)	50.2 (30.4 – 71.4)
%Spent in bouts 31-60 minutes	16.2 (10.6 – 20.3)	n.a.	13.4 (9.5 – 19.8)	16.5 (12.7 – 20.5)
Time in bouts 31-60 minutes	128.4 (88.0 – 147.8)	n.a.	130.2 (62.7 – 162.3)	125.6 (91.5 – 149.8)
%Spent in bouts >60 minutes	42.4 (29.2 – 67.1)	n.a.	72.9 (56.4 – 82.2)	34.6 (18.7 – 43.6)
Time in bouts >60 minutes	323.5 (191.4 – 528.3)	n.a.	541.6 (520.2 – 692.4)	223.8 (132.6 – 361.2)
Bout length	22.7 (10.0 – 34.3)	n.a.	43.2 (30.2 – 64.3)	11.5 (8.5 – 23.0)
W50	24.0 (6.0 – 77.0)	n.a.	81.0 (27.3 – 135.8)	9.0 (5.5 – 24.0)

**Bout outcomes are derived only from sensors that measured every minute*

Home-based data				
	**All**	**Type 1**	**Type 2.1**	**Type 2.2**
**Movement during the day** ***Median (IQR)***	***N=19***	***N=5***	***N=3***	***N=11***
Wear time	796.6 (707.8 0 959.0)	707.8 (603.1 – 870.6)	n.a.	796.6 (724.0 – 959.0)
%Spent in SB	90.5 (79.9 – 97.7)	99.0 (98.2 – 100.0)	n.a.	86.9 (76.0 – 90.5_
Minutes in SB	704.2 (600.4 – 896.4)	704.2 (599.0 – 862.1)	n.a.	675.1 (597.9 – 754.7)
%Spent in LPA	9.5 (2.3 – 19.8)	1.0 (0.0 – 1.8)	n.a.	13.0 (9.5 – 23.9)
Minutes in LPA	90.6 (16.7 – 177.3)	3.6 (0.4 – 12.3)	n.a.	149.9 (90.6 – 186.0)
%Spent in MVPA	0.0 (0.0 – 0.2)	0.0 (0.0 – 0.0)	n.a.	0.0 (0.0 - 0.2)
Mean minutes in MVPA	0.3 (0.0 – 1.6)	0.0 (0.0 – 0.0)	n.a.	0.3 (0.1 – 1.9)
PAM score	8.8 (5.3 – 14.9)	5.3 (4.7 – 7.9)	**n.a.**	12.6 (7.9 – 15.1)
**Bouts***	***N= 14***	**n.a.**	***N=3***	***N= 11***
Number of bouts	55.5 (37.5 – 78.8)	n.a.	n.a.	57.3 (42.5 – 77.2)
%Spent in bouts 31-60 minutes	17.7 (11.4 – 21.9)	n.a.	n.a.	19.1 (14.8 – 21.5)
Time in bouts 31-60 minutes	147.4 (102.6 – 203.1)	n.a.	n.a.	150.0 (114.2 – 177.4)
%Spent in bouts >60 minutes	31.8 (187 – 40.4)	n.a.	n.a.	25.5 (15.2 – 38.8)
Time in bouts >60 minutes	275.6 (165.8 – 448.1)	n.a.	n.a.	172.5 (160.8 0 342.5)
Bout length	14.9 (9.4 – 20.3)	n.a.	n.a.	14.7 (7.9 – 17.8)
W50	30.0 (19.0 – 46.3)	n.a.	n.a.	25.0 (10.0 – 38.0)

**Bout outcomes are derived only from sensors that measured every minute*
